# On-demand optogenetic activation of human stem-cell-derived neurons

**DOI:** 10.1038/s41598-017-14827-6

**Published:** 2017-10-31

**Authors:** Simon D. Klapper, Evelyn J. Sauter, Anka Swiersy, Max A. E. Hyman, Christian Bamann, Ernst Bamberg, Volker Busskamp

**Affiliations:** 1 Center for Regenerative Therapies Dresden (CRTD), Technische Universität Dresden, Dresden, Germany; 2 Max Planck Institute of Biophysics, Frankfurt, Germany

## Abstract

The widespread application of human stem-cell-derived neurons for functional studies is impeded by complicated differentiation protocols, immaturity, and deficient optogene expression as stem cells frequently lose transgene expression over time. Here we report a simple but precise Cre-loxP-based strategy for generating conditional, and thereby stable, optogenetic human stem-cell lines. These cells can be easily and efficiently differentiated into functional neurons, and optogene expression can be triggered by administering Cre protein to the cultures. This conditional expression system may be applied to stem-cell-derived neurons whenever timed transgene expression could help to overcome silencing at the stem-cell level.

## Introduction

Recent efforts in generating human neurons from pluripotent stem cells^1^ *in vitro* mean that it is now easier to study human neuronal development, maturation, function, and pathology. Human stem cells have a high proliferation rate and are therefore an abundant cellular resource. Furthermore, these cells can easily be manipulated and selected at the stem-cell level before being differentiated into the desired cell types including neurons^2^. This is an advantage over primary human neurons, which are postmitotic and therefore do not divide. In addition, the quality and composition of primary neurons can be highly variable due to batch effects. Still, many stem-cell differentiation protocols also suffer from heterogeneous and immature neuronal populations after weeks, or even months, of culture^3^. However, there has recently been a steady emergence of advanced protocols based on small molecules^4^ or transcription factor induction^3,5,6^: these protocols have improved differentiation time, yield, purity and maturation to electrically active neurons^7,8^.

Neuronal activity in stem-cell-derived neurons can be activated by conventional electrophysiological stimulation or by an attractive alternative, called optogenetics^9^, which has recently become the gold standard. Optogenes are DNA-encoded light-sensitive ion channels or pumps that change the cellular polarization upon light activation and thereby excite or inhibit neuronal activity^10^. Regarding temporal and spatial resolution, optogenetics has considerably elevated the standard for investigating electrically excitable cells and their networks *in vivo* as well as *in vitro* ^11^. However, optogenetic applications *in vitro* are severely limited, as obtaining the expression levels required for functional studies is not trivial. Specifically, stem cells frequently lose transgene expression over time^12,13^. Generating monoclonal transgenic human embryonic stem-cell (ESC) lines in which the optogene is driven by a ubiquitous^14^ or a neuronal promoter^15,16^ is an option, but has turned out to be laborious because generating and testing monoclonal lines may easily take several weeks. Furthermore, the long-term stability of expression in these cell lines is unclear as it has not been assessed over multiple passages. We have observed that the expression of a genomically-integrated optogene driven by a neuronal promoter in human induced pluripotent stem cells (iPSCs) was low (Supplementary Fig. 1a,b), and vanished after a few passages. Commonly used neuronal promoter elements are not specific in human iPSC-derived neurons: they are already active in stem cells (Supplementary Fig. 1c)^5^. This baseline expression of optogenes probably leads to their silencing in stem cells. Alternatively, differentiated neurons can be optogenetically tagged by viral gene delivery^3,7^. However, *de novo* transduction is required for every experiment, and may result in variable efficiency and low transduction rates^7^. Hence, stable transgenic stem-cell lines, in which optogene expression can be precisely triggered, will be an advanced and user-friendly system, allowing more widespread application of optogenetics in stem-cell-derived neurons and neural organoids^17,18^.

## Results

**Figure 1:**
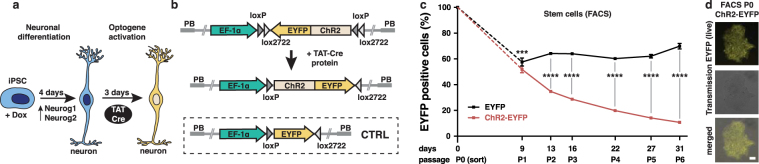
Controlling optogene expression in conditional human iPSC lines. (**a**) Scheme of rapid iNGN cell differentiation and optogenetic activation. (**b**) Schemes of the double-floxed inverse open reading frame under the EF1α promoter (eDIO) ChR2-EYFP construct (top) and its inversion by TAT-Cre recombinase (middle). The EYFP construct (bottom) served as a control (CTRL). (**c**) Flow cytometry of EYFP-positive cells from iNGN (iPSC) cultures treated with TAT-Cre over subsequent passages (P). The fraction of fluorescent ChR2-EYFP cells decreased significantly compared to EYFP cells (P1: p = 0.005, P2: p < 0.0001, P3: p < 0.0001, P4: p < 0.0001, P5: p < 0.0001, P6: p < 0.0001, 2-way ANOVA with Sidak method, n = 3 culture wells). Error bars indicate standard deviation. (**d**) Example live images of TAT-Cre-treated uninduced iNGN (iPSC) colonies after FACS. Scale bars, 100 µm. *p < 0.05, **p < 0.01, ***p < 0.001, ****p < 0.0001, n.s., not significant.

The precision of the Cre-loxP system in genetic manipulations has been known for decades^19^. We have adapted this system to human iPSCs, in which neurogenesis can be induced rapidly (iNGN cells, Fig. 1a)^5^. We generated Cre-inducible conditional ChR2-EYFP and EYFP expression cassettes^20^, so-called double-floxed inverse open reading frame (DIO) constructs that were genomically integrated by the piggyBac transposon system^21^ into iNGN cells (Fig. 1b and Supplementary Fig. 1d). Administering 0.59 µM Cre protein (TAT-Cre), which is available commercially, directly to the culturing medium for 2 h was found to be most efficient for transgene activation by inverting the open reading frame (Supplementary Fig. 1e). First, we triggered transgene expression in iPSCs (uninduced iNGNs), sorted fluorescent cells, and followed their fraction over six flow cytometry passages (Fig. 1c and Supplementary Fig. 1f–i). Within the first passage, which spanned 9 days, the levels of fluorescently labelled ChR2-EYFP cells dropped to 51.8 ± 1.5% and EYFP cells to 57.6 ± 1.8% (Fig. 1c), although initial colonies consisted of only fluorescently labelled cells (Fig. 1d). ChR2-EYFP expression decreased significantly over six passages to 10.7 ± 0.3%, whereas EYFP expression remained stable and ranged between 60.2% and 69.9% (Fig. 1c). Here, our data highlight the instability of membrane-bound optogene expression in stem cells. In contrast, we observed stable expression of a cytosolic fluorescent protein in stem cells over six passages. We did not detect any chromosomal translocations after Cre-induction in ChR2-EYFP cells (Supplementary Fig. 1j), suggesting that Cre activity occurred only locally at the integrated DIO cassettes.

**Figure 2:**
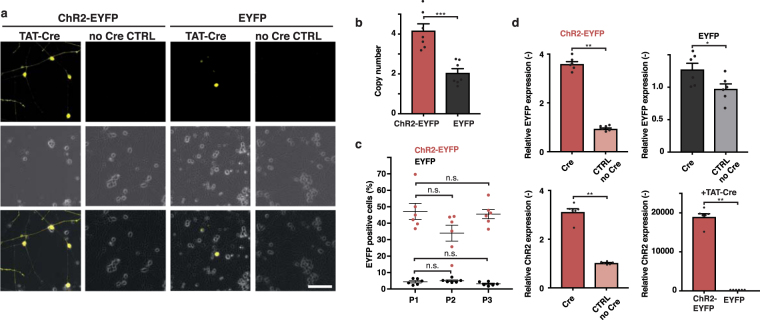
Optogene activation in stem-cell-derived neurons. (**a**) Example live images of TAT-Cre-treated induced iNGN neurons and no-Cre controls at 7 dpi. (**b**) Number of integrated copies in iNGN-eDIO-ChR2-EYFP and iNGN-eDIO-EYFP cells by qRT-PCR. Copy number was significantly lower in EYFP cells (p = 0.0002, unpaired t-test, n = 7 culture wells). (**c**) Quantification of neurons expressing EYFP and ChR2-EYFP at 7 dpi. Neurons were differentiated after each 3 subsequent passages and activated by Cre administration at 4 dpi. The EYFP signal did not change with passaging. (ChR2-EYFP P2: p = 0.9804, P3: p = 0.2510, EYFP P2: p = 0.9990, P3: p = 0.8695, 2-way ANOVA with Sidak method versus P1, n = 6 culture wells). (**d**) qRT-PCR expression levels of EYFP (top) and ChR2 (bottom), TAT-Cre-treated vs. vehicle-treated. Relative EYFP (ChR2-EYFP, top left: p = 0.0022; EYFP, top right: p = 0.0411) and ChR2 (bottom left: p = 0.0022) expression was significantly higher after TAT-Cre treatment compared to no-Cre CTRL. ChR2 expression was significantly higher in ChR2-EYFP than in EYFP cells (p = 0.0022) after TAT-Cre treatment (all Mann-Whitney test, n = 6 culture wells). Scale bars, 100 µm. *p < 0.05, **p < 0.01, ***p < 0.001, ****p < 0.0001, n.s., not significant.

Next, we activated ChR2-EYFP and EYFP expression at 4 days post neuronal induction (dpi) and analysed fluorescently labelled neurons by live-cell microscopy at 7 dpi (Fig. 2a). About 43.4 ± 2.7% of the neurons expressed ChR2-EYFP and, probably due to lower copy numbers, 4.6 ± 0.4% expressed EYFP (Fig. 2c). The conditional iNGN cells were not monoclonal: they had 4.2 ± 0.3 copies (iNGN-eDIO-ChR2-EYFP) and 2.1 ± 0.2 copies (iNGN-eDIO-EYFP) of the piggyBac cassettes (Fig. 2b). We passaged iNGN-eDIO-ChR2-EYFP and iNGN-eDIO-EYFP cells for multiple passages. At each passage, we differentiated a fraction of the cells into neurons, activated optogene expression at 4 dpi by Cre, and quantified fluorescently labelled cells at 7 dpi. We obtained stable Cre activation rates over multiple consecutive passages in neurons (Fig. 2c and Supplementary Fig. 2a). We used quantitative RT-PCR to measure ChR2 and EYPF expression levels. We detected ChR2 and EYFP transcripts in the non-Cre-treated iNGN-eDIO-ChR2-EYFP and EYFP samples (no-Cre CTRL), suggesting that the reverse complementary transcripts were also transcribed. However, Cre induction resulted in significant increases in expression of ChR2 (3.1±0.1-fold) and EYFP (3.6 ± 0.1-fold) in iNGN-eDIO-ChR2-EYFP cells, and 1.3±0.1-fold EYFP induction in iNGN-eDIO-EYFP cells (Fig. 2d). Comparing iNGN-eDIO-ChR2-EYFP to iNGN-eDIO-EYFP, ChR2 expression levels increased upon Cre administration 18,918 ± 853-fold (Fig. 2d). Expression levels of the neuronal marker MAP2 were not significantly different between Cre-activated and control samples (Supplementary Fig. 2b, c), suggesting regular neuronal induction. In summary, the expression of activated membrane-bound ChR2-EYFP in human iPSCs decreased significantly over a few passages, but was robust when activated in differentiated neurons.

**Figure 3:**
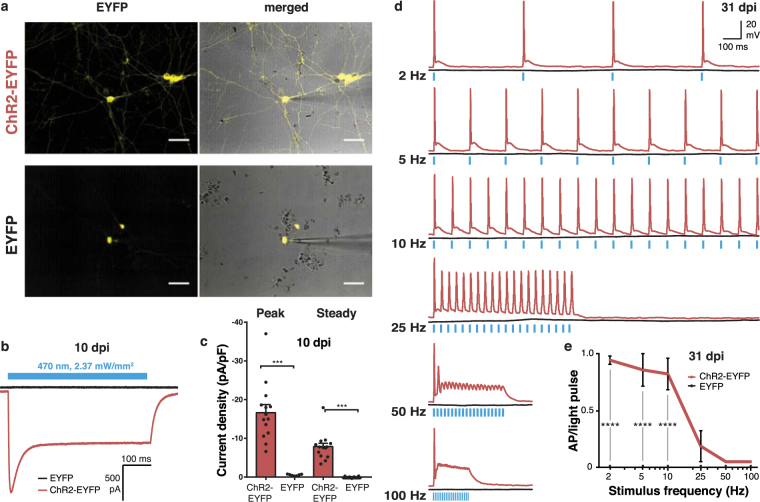
Functional recordings from optogenetically tagged neurons that were activated at 7 dpi. (**a**) Example live images of iNGN-eDIO-ChR2-EYFP (top) and iNGN-eDIO-EYFP (bottom) after patch-clamp recordings at 10 dpi. EYFP fluorescence (yellow, left) and overlay with transmission image (right) are shown. (**b**) Example traces of voltage-clamp recordings at 10 dpi. iNGN cells were excited using 500 ms light pulses. (**c**) Quantification of peak and steady current density at 10 dpi. Current densities were significantly higher in neurons expressing ChR2-EYFP than in those expressing EYFP (peak: p < 0.0001, steady: p < 0.0001, Mann-Whitney test, n = 14 cells from 4 cover slips and 2 preparations). (**d**) Example traces of current clamp recordings at 31 dpi. iNGN cells were excited using 20 light pulses of 1 ms with increasing frequencies. (**e**) Quantification of evoked action potentials (AP) versus number of stimulus pulses at 31 dpi for different stimulus frequencies. APs per light pulse were significantly higher in ChR2-EYFP than in EYFP expressing neurons for up to 10 Hz (2 Hz: p < 0.0001, 5 Hz: p < 0.0001, 10 Hz: p < 0.0001, 25 Hz: p = 0.3391, 50 Hz: p = 0.9969, 100 Hz: p = 0.9969, 2-way repeated measures ANOVA with Sidak method, n = 7 cells from 3 cover slips). Scale bar, 100 µm. *p < 0.05, **p < 0.01, ***p < 0.001, ****p < 0.0001, n.s., not significant.

Next, we assessed the optical control of neuronal function in differentiated iNGN cells in astrocyte co-cultures^22^ at 10 dpi and 31 dpi, in which Cre had been applied at 7 dpi (Supplementary Fig. 2d). We performed whole-cell patch-clamp recordings from fluorescently labelled neurons (Fig. 3a). Robust photocurrents were evoked at 10 dpi by 470 nm light stimulation (2.37 mW/mm^2^, Supplementary Fig. 2e) in neurons expressing ChR2-EYFP but not EYFP (Fig. 3b). Photocurrent densities peaked at −16.7 ± 2.0 and 0.01 ± 0.17 pA/pF in ChR2-EYFP and EYFP cells, respectively (Fig. 3c). Steady photocurrent densities settled at −7.8 ± 0.9 and 0.003 ± 0.03 pA/pF. The average cell capacitance that corresponds to the cellular outgrowth was not different between neurons expressing ChR2-EYFP and EYFP (Supplementary Fig. 2h), suggesting similar neuronal growth and cell sizes. To evoke action potentials, we performed functional recordings from neurons at 31 dpi (Fig. 3d) which resulted in robust triggering of action potentials in neurons expressing ChR2-EYFP, but not in those expressing EYFP (Fig. 3e). 1 ms light pulses (4.75 mW/mm^2^, Supplementary Fig. 2e) resulted in single action potentials with efficiencies of between 82.1% and 94.3% for up to 10 Hz (Fig. 3e). At higher frequencies, subsequent light pulses did not robustly trigger repeating action potentials. The capacitance (Supplementary Fig. 2i) and maximum action potential frequency (Supplementary Fig. 2j) evoked by current injections did not differ between neurons expressing ChR2-EYFP and EYFP, suggesting that the neurons analysed were at similar developmental stages at 31 dpi. We also functionally tested the inhibitory optogene Jaws^23^ (Supplementary Fig. 2f) for neuronal silencing using whole-cell patch-clamp recording with the light source at 580 nm (5.22 mW/mm^2^, Supplementary Fig. 2e). Activation of Jaws-EGFP at 7 dpi yielded 1.2 ± 0.18 peak and 1.1 ± 0.2 pA/pF steady photocurrents at 10 dpi (Supplementary Fig. 2g). Here, we show that Cre-activated optogene expression in stem-cell-derived neurons allows for robust optical control of neuronal activity for more than three weeks after activation. The required light levels were relatively low (Supplementary Fig. 2e), suggesting that the range of optogene expression was optimal for functional activation.

## Discussion

We integrated the conditional DIO Cre-loxP system into human iPSCs to control optogene expression precisely and on demand after neuronal differentiation of human iPSCs. Our data indicate that optogenes, which were genomically integrated as inverted open reading frames, are tolerated and excluded from silencing in stem cells. From an *in-vivo* perspective, using the Cre-loxP system for optogene expression control sounds trivial. From a stem-cell perspective, it represents a technical advance towards the efficient generation of light-controllable human neurons. Previously, the Cre-loxP system has been successfully used to remove or activate reporter and selection cassettes in human ESCs and neural precursors^24^. Here we demonstrate its application in post-mitotic stem-cell-derived neurons in facilitating optogenetic activation and silencing. PiggyBac vector generation, electroporation and selection for genomic integration took less than three weeks compared to the average of several months which is needed for screening genome-edited monoclonal lines bearing transgene cassettes. Administering non-viral TAT-Cre recombinase to the culturing media places the technique at biosafety level 1: this is therefore a user-friendly system for functional human neuronal studies *in vitro*, from single-cell to network level, by high-throughput multi-electrode arrays (Supplementary Fig. 2k). Stem-cell-derived neurons, which can be activated or silenced by light, can serve as powerful and easy-to-use human model systems to complement or follow up *in-vivo* studies with biomedical foci. For example, they can serve as connectors from rodent proof-of-concept combined optogenetic and cell replacement studies^25^ to preclinical models using human stem-cell-derived neurons to accelerate translation.

Obtaining 40% ChR2-EYFP-positive cells is more advantageous than the levels obtained by adeno-associated-viral delivery (1%)^7^, and is sufficient for neuronal network studies that would be masked if all cells were triggered by light. Activation efficiencies may be further improved by using modified recombinant Cre versions^26^ or by using tamoxifen-dependent Cre recombinases^27^ which can be included in the piggyBac vectors: this would result in an all-in-one conditional and small-molecule-triggered expression system.

Besides optogenetics, the eDIO system represents a powerful control unit for gene-of-interest expression whenever leaky expression cannot be tolerated within stem cells or certain differentiation states. Furthermore, revealing the biological mechanisms of how and which transgenes are silenced in stem cells is of high interest in the stem-cell community, and can be studied in our conditional human iPSC lines. FACS resulted in an initial decrease of both ChR2-EYFP and EYFP expression within the first passage, which shows that sorting stem cells negatively influences transgene expression. ChR2-EYFP expression further decreases over six passages, whereas EYFP levels remain unaltered, suggesting that the type of ectopically expressed protein used influences stable expression over multiple passages. It is noteworthy that lentiviral transduction shows the expression of cytosolic fluorescent reporter proteins to be stable in human stem cells and differentiated neurons^28^: this is in line with robust EYFP expression in iNGN cells over several passages.

Altogether, we have demonstrated that combining robust, existing gene-control and stem-cell technologies provides powerful tools for enabling timed transgene expression and optogenetic applications in human stem-cell-derived neurons in a user-optimised fashion.

## Methods

### Plasmid generation

The piggyBac vector backbone PB-TRE-dCas9-VPR^29^ was a gift from George Church (Addgene plasmid, 63800). All promoter elements and open reading frames between the 5′ core insulator and the SV40 polyA were removed and replaced with a PCR-amplified ubiquitin C promoter-blasticidin (Bla) cassette from vector pLV-TRET-hNgn1-UBC-Bla^5^ (Addgene plasmid, 61473). All restriction enzymes were purchased from New England Biolabs. The PCR primers were designed to have 20-nucleotide (nt) homologous overhangs to the vector backbone. PCR products were introduced into the piggyBac vector using Gibson Assembly (GA) cloning^30^ with two GA mastermixes made in-house. The plasmid DNA was transformed in chemically competent bacteria (One Shot® Stbl3™, Thermo Fisher Scientific) following the manufacturer’s guidelines. The human synapsin I promoter driving a DsRedExpress cassette from pLV-hSyn-RFP^31^ (gift from Edward Callaway, Addgene plasmid, 22909), together with a downstream WPRE-bovine growth hormone polyadenylation signal (BGH-pA), were further added to obtain piggyBac-Syn-DsRed-BGH-UBC-Bla.

### piggyBac-Syn-CatCh-EGFP-UBC-Bla

The ChR2 derivate CatCh^32^, fused to EGFP, was PCR-amplified and cloned by GA into the BamHI-linearised piggyBac-Syn-DsRed-BGH-UBC-Bla backbone to replace DsRed: this resulted in piggyBac-Syn-CatCh-EGFP-UBC-Bla.

### Double-floxed inverse open reading frame (eDIO) constructs

The piggyBac-Syn-DsRed-BGH-UBC-Bla vector backbone was linearised using SfiI/BamHI to excise Syn-DsRed. The EF1α-DIO cassette (eDIO), including hChR2(E123A)-EYFP^33^ (ChR2-EYFP), was PCR-amplified using 20-nt overhang GA primers from pAAV-EF1α-DIO-hChR2(E123A)-EYFP^20^, which was kindly donated by Karl Deisseroth (Addgene plasmid, 35507). The piggyBac-eDIO-ChR2-EYFP construct was assembled by GA. We designed a general cloning strategy in which the ChR2-EYFP cassette was excised by AscI/NheI and replaced by PCR-amplified optogenes and fluorescent reporters. The primers were designed to have a 6-nt spacer at the end, followed by 5′ NheI and 3′ AscI adapters and 21 nt that were sequence-specific for PCR amplification. The open reading frames were placed in reverse complementary orientation. PCR products were gel-extracted (2% E-Gel® EX, Thermo Fisher Scientific), purified (MinElute Gel Extraction Kit, Qiagen) and subsequently digested with AscI/NheI. The vector backbone and insert were ligated (DNA Ligation Kit, Mighty Mix, TaKaRa) and transformed in chemically competent bacteria (One Shot® Stbl3™, Thermo Fisher Scientific) following the manufacturer’s guidelines. The following eDIO constructs were generated: the EYFP sequence was PCR-amplified from pAAV-EF1α-DIO-hChR2(E123A)-EYFP^20^ (Addgene plasmid, 35507): Jaws-EGFP was PCR-amplified from fck-Jaws-KGC-GFP-ER2^23^, a gift from Edward Boyden (Addgene plasmid, 65012).

### iNGN cell culture

The iNGN cell line^5^ at passage number 44 was a gift from George Church. This cell line is part of the ENCODE catalogue (https://www.encodeproject.org, accession number: ENCBS369AAA). The cells were cultured, propagated and cryopreserved as previously described^5^. Cells at maximum passage number 55 were used in this study and checked for mycoplasma contamination in four-week intervals using the Universal Mycoplasma Detection Kit ATCC® 30–1012 K™ (ATCC, 30–1012 K).

### Nucleofections

The 4D-Nucleofector™ System (Lonza) was used to electroporate piggyBac and transposase vectors into iNGN cells in suspension (X-Unit, P3 Primary Cell 4D-Nucleofector® X Kit L, program CB-156) following the manufacturer’s guidelines. By default, 10 µg transposon DNA and 2 µg transposase vector (System Biosciences) were applied in less than 10 µl volume to 800k iNGN cells. After nucleofections, cells were kept under constant 20 µg/ml blasticidin (Thermo Fisher Scientific, A1113903) selection.

### Neuronal cultures

The iNGN cells were differentiated using 0.5 µg/ml doxycycline (Sigma, D9891) in mTeSR1 medium (Stem Cell Technologies, 05850), supplemented with penicillin/streptomycin (Thermo Fisher Scientific, 15140122) as previously described^5^. At 4 dpi, 5 µM Ara-C (Sigma, C6645) was added to the culture to remove occasional undifferentiated cells. To enhance long-term survival and maturation, the neurons were re-seeded at 5 dpi onto thoroughly cleaned cover slips coated in PDL (0.1 mg/ml, Merck Millipore, A-003-E) and laminin (50 µg/ml, Sigma, L2020), and equipped with paraffin feet (Polysciences, 24364) as previously described^22^. After 2 h, cover slips with differentiated iNGN cells were placed upside down into culture wells containing 80% confluent rat astrocytes (Thermo Fisher Scientific, N7745). For multi-electrode array (MEA) recordings, neurons were re-seeded at 5 dpi onto MEAs (Multi Channel Systems, 60PedotMEA200/30iR-Au-gr) coated in PDL (0.1 mg/ml) and laminin (50 µg/ml). To co-culture the neurons on the MEAs with astrocytes, rat astrocytes were seeded onto PDL-coated (0.1 mg/ml) cover slips (15 mm diameter), equipped with paraffin feet and grown to 80% confluency. 2 h after re-seeding of neurons, cover slips with astrocytes were placed upside down onto the MEAs. Astrocytes were maintained according to manufacturer’s instructions using astrocyte medium (Thermo Fisher Scientific, A1261301) with penicillin/streptomycin. One day before re-seeding of neurons, astrocytes were washed three times with DPBS with calcium and magnesium (Thermo Fisher Scientific, 14287–080). BrainPhys medium (StemCell Technologies, 05790), supplemented with penicillin/streptomycin, SM-1 (StemCell Technologies, 05711), N-2 (Thermo Fisher Scientific, 17502048), and 0.2 µM ascorbic acid (Sigma, A0278) were added. Every 7 days, 50% of the supplemented BrainPhys medium was exchanged and volume loss due to evaporation was compensated with dH_2_O (CRTD media kitchen).

For qRT-PCR and quantification experiments, cells were directly differentiated in PLL (40 µg/ml, Sigma, P6407) and laminin-coated wells. On 4 dpi, the cells were induced with TAT-Cre and the medium was changed to 50% BrainPhys medium supplemented with SM-1, N-2, 0.2 µM ascorbic acid, 20 ng/ml BDNF (Peprotech, 450-02), 20 ng/ml GDNF (Peprotech, 450-10), 1 mM cAMP (Sigma, D0627), and penicillin/streptomycin.

### Cre administration

TAT-Cre protein (Merck Millipore, SCR508; Bio-Connect Services B.V., EG-1001) was diluted to 20 units/ml (~0.59 μM) in cell culture supernatant and added to the cells for 2 h at 37 °C, 5% CO_2_ before washing with DPBS with calcium and magnesium (Thermo Fisher Scientific, 14287-080); fresh culture media was then added. For astrocyte co-cultures, a mixture of fresh media and supernatant was used. For no-Cre CTRL conditions, only the TAT-Cre buffer was used. Typically, in stem cells an average of 4.9% of ChR2-EYFP cells and 1.3% of EYFP cells were EYFP-positive, whereas in neurons around 42% of ChR2-EYFP cells and 4% of EYFP cells were EYFP-positive after Cre administration (Supplementary Figs 1g and 2c).

### Imaging

Images were acquired at 20× magnification using the EVOS™ FL Imaging System (Thermo Fisher Scientific, AMF4300) or the Axio Oberserver.Z1 (Zeiss) of the Light Microscopy Facility, a core facility of BIOTEC/CRTD at the Technische Universität Dresden. Data were analysed using the Fiji Software^34^. The number of EYFP-positive neurons was quantified by manual counting. Images of patch-clamped neurons were acquired after recording using an analogue IR camera (TILL Photonics VX55) in combination with a frame grabber (Imaging Source DFG/USB2pro), and saved on a PC using Micro-Manager 1.4^35,36^.

### Flow cytometry

Cells were dissociated using TrypLE Express (Thermo Fisher Scientific, 12604013), washed in 1× DPBS without calcium and magnesium (Thermo Fisher Scientific, 14190169) and resuspended in FACS buffer containing 1× DPBS + 10% foetal bovine serum (Thermo Fisher Scientific, 26140-079) + 10 mM EDTA (Thermo Fisher Scientific, 15575-038). To sort stem cells, samples were collected on a BD FACSAria III flow cytometer. FSC-A/SSC-A were used to exclude debris and apoptotic cells; doublets were excluded by using FSC-W/FSC-H; the EYFP + sorting gate was set using SSC-A/FITC-A (Supplementary Fig. 1i). Uninduced cells were used as a negative control. The abundance of the relevant cell populations within the post-sort fraction was confirmed by an immediate post-sort analysis of 100–1000 cells at the sorter including a viability dye (PI): ≥95% of cells were viable EYFP-positive cells. Stem cells were analysed on a BD LSR II flow cytometer using the BD FACSDiva software v8.0.2. Debris and apoptotic cells were excluded by using FSC-A/SSC-A; singlets were identified by using FSC-H/FSC-A and SSC-W/SSC-H; the EYFP + analysis gate was set using SSC-A/YFP-A (Supplementary Fig. 1h,i). The quantification of EYFP-positive neurons by flow cytometry was unreliable, and images were therefore counted manually.

### Karyotyping

The analysis was done on GTG-stained metaphase preparations with a mean resolution of 200 bands per haploid chromosome set (comparable to human bone marrow quality). Sub-microscopic changes (microdeletions/microduplications) and changes smaller than 10 Mb cannot be excluded by this method; mosaicism is only detected and mentioned in the karyotype formula if chromosomal losses appear at least 3 times or structural aberrations and gains are observed at least two times. Composite karyotype [cp] was given regularly from 20 metaphase spreads each.

### Quantitative RT-PCR

1,000,000 cells were plated in Matrigel-coated 6-well plates and induced with doxycycline for four days. At 4 dpi, 0.59 µM TAT-Cre were administered for 2 h and, subsequently, the medium was replaced with 50% BrainPhys medium. At 7 dpi, total RNA was extracted automatically using the RNeasy Mini Kit (Qiagen, 74104) in combination with a Qiacube (Qiagen, 9001885), and cDNA was synthesised using the High-Capacity cDNA Reverse Transcription Kit (Thermo Fisher Scientific, 4368814) according to the manufacturer’s instructions. PCR amplification was performed using Power SYBR^®^ Green PCR Master Mix (Thermo Fisher Scientific, 4367659) on a StepOnePlus™ Real-Time PCR System (Applied Biosystems) by denaturation at 95 °C for 10 min and 40 cycles at 95 °C for 50 s and 60 °C for 1 min, followed by a melting curve to confirm the amplification specificity. Six biological replicates were used for each TAT-Cre and no-Cre CTRL sample, and values were normalised on ACTB expression levels. The data were analysed using the ΔΔC_T_ method^37^.

### Cells-to-C_T_ 1-Step quantitative RT-PCR

20,000 iNGN-eDIO-ChR2-EYFP cells were plated in Matrigel-coated 96-well plates and induced with doxycycline for four days. At 4 dpi, 200 µl mTeSR1 with varying TAT-Cre concentrations (0.00, 0.06, 0.12, 0.29, 0.39, 0.59, 1.17 and 2.93 µM) were applied for 2 h and, subsequently, the medium was replaced with plain mTeSR1. At 6 dpi, the cells (<100,000 cells per sample) were lysed using the Power SYBR Green Cells-to-C_T_^TM^ 1-Step Power SYBR™ Green Kit (Ambion, A25601), and RNA samples were processed for quantitative RT-PCR according to the user manual, using a StepOnePlus™ Real-Time PCR System (Applied Biosystems). Six biological replicates were used for each TAT-Cre concentration to detect ChR2 expression levels: these replicates were normalised on ACTB. Diluted cell lysates served as no reverse transcription (no-RT) controls and resulted, on average, in C_T_ values > 11.9 (ACTB primer sets, Table 1) compared to the corresponding RT samples. Still, we detected ChR2 signals that probably stemmed from genomic DNA content. The data were analysed using the ΔΔCT method^37^.

**Table 1:** qRT-PCR primer sequences.

ACTB fwd	CCTGGATAGCAACGTACATGG
ACTB rev	ACCTTCTACAATGAGCTGCG
EYFP fwd	CAACAGCCACAACGTCTATATCATG
EYFP rev	ATGTTGTGGCGGATCTTGAAG
ChR2 fwd	ATGGCATGGCTGTTTTTCGT
ChR2 rev	CTCAGGACGCCAAAACCTTC
MAP2 fwd	CACAGTGGAGGAAGCAGCA
MAP2 rev	GGGCTCTTGGTTACTCCGTC

### Copy number quantification

Genomic DNA was extracted from cell pellets using the DNeasy Blood and Tissue Kit (Qiagen, 69504) in combination with a Qiacube (Qiagen, 9001885). Quantitative PCR was performed using the Power SYBR^®^ Green PCR Master Mix (Thermo Fisher Scientific, 4367659) on a StepOnePlus™ Real-Time PCR System (Applied Biosystems) using the primers provided with the piggyBac copy number kit (System Biosciences, PBC100A-1). The number of integrated copies was determined according to the manufacturer’s protocol.

### Electrophysiology

During the experiment, single cover slips with neurons were kept in filtered (0.2 µm) extracellular solution of the following composition (mM): NaCl 130 (Sigma, S3014), HEPES 20 (Thermo Fisher Scientific, 15630056), glucose 10 (CRTD media kitchen), KCl 5 (Merck, 1049360250), CaCl_2_ 2.5 (CRTD media kitchen), MgCl_2_ 1 (CRTD media kitchen), pH 7.3 with NaOH (Merck, 1064950250). Neurons expressing ChR2-EYFP, EYFP, or Jaws-EGFP were identified under an upright microscope (Zeiss Examiner.A1 Axio) equipped with a water-immersion 20× objective (Zeiss, 420957-9900). For stimulation, the microscope optics were fed with a Spectra4-LCR-XA (Lumencor) LED light source that was triggered by TTL pulses, and the microscope’s filter cube was equipped with a silver-coated mirror (Thor labs, PFR10-P01). Applied light power levels at the focal plane were measured using a PM100 power meter (Thor labs). Power settings of 5% or 10% (Fig. S2b) were sufficient for robust photocurrent induction. The 513 or 470 nm channels, in combination with YFP (Zeiss, 38) or GFP filter cubes (Zeiss, 46), were used to visualise EYFP or EGFP fluorescence, respectively. Neurons with high EYFP or EGFP expression were selected for single-cell patch-clamp using whole-cell configuration. Microelectrodes with resistances of ~6 MΩ were pulled from borosilicate glass (Science Products, GB150EFT-8P) with a P-1000 programmable horizontal puller (Sutter Instruments), and contained a filtered (0.2 µm) intracellular solution of the following composition (mM): KCl 130, HEPES 20, EGTA 10 (Merck, 324626), CaCl_2_ 0.25, pH 7.3 with KOH. The head stage was attached to a digitally controlled micro-manipulator (Luigs & Neumann SM-7) and connected to a multi-clamp 700 amplifier (Molecular Devices): analogue electrophysiological data was low-pass filtered at 6 kHz and subsequently converted to digital values at a sample rate of 20 kHz using a Digidata 1440 A (Molecular Devices). Data were transferred to a PC using pClamp software (10.3, Molecular Devices) and analysed using Clampfit software (10.3, Molecular Devices) and custom Python (python.org) code with the neo package (https://pythonhosted.org/neo). Pipette capacitance was always compensated for. For each neuron, several −10 mV pulses were applied after opening the cell to measure the capacitance in voltage-clamp mode at −60 mV, and subsequently averaged. Capacitance was calculated as the integral of the capacitive current (carried charge) at offset of pulse, divided by the pulse amplitude. Cell capacitance was compensated for before further voltage clamp recordings. To measure photocurrents, 1 µM tetrodotoxin (Tocris, 1078) was added to the extracellular solution to block voltage gated sodium currents. Neurons were held at −100 mV; 500 ms pulses of 470 nm (ChR2/EYFP) or 580 nm (Jaws) were used (Supplementary Fig. 2e). Peak and steady-state responses were analysed at the end of the light pulse. Spiking behaviour was assessed in current-clamp mode and constant current was injected to hold cells at around −70 mV. Spikes were then evoked by 20 × 1 ms light pulses at increasing frequencies from 2 to 100 Hz with a 5 s break between the pulse trains. Action potentials were detected using a threshold at −10 mV, and the ratio of action potentials per light pulse was calculated. To find a neuron’s maximum spike frequency, 500 ms increasing current steps were injected from −30 to 150 pA in 10 pA steps. All membrane voltages were corrected for a liquid junction potential, which was calculated to be 0.4 mV. No cover slip was measured for longer than 1 h.

An MEA1060-Inv-BC system (Multi Channel Systems) with the heating plate set to 37 °C was used for MEA recordings. For optical stimulation, the MEA head stage was placed onto the stage of an inverted microscope (Nikon, Eclipse Ti) and the electrode field was aligned to the field of view of a 10× objective. Light stimulations were done similarly to patch clamp experiments using the Spectra4 light source fed through the microscope’s optics. Ten 500 ms light pulses were applied from the 470 nm channel (4.15 mW/mm^2^), with a 5 s inter-pulse interval. Recordings were done in culture medium with astrocytes present; MEAs were kept outside the incubator for no longer than 15 minutes. Raw electrode data was acquired via MC_Rack software (Multi Channel Systems) at 25 kHz sample rate. Data was analysed using a custom Matlab (Mathworks) script. Spikes were detected in filtered data (200 Hz high pass and 2 kHz low pass) by setting a threshold to 5 times the standard deviation outside of stimulation pulses.

### Data and Code availability statement

The datasets generated during the current study are available from the corresponding author on reasonable request. All custom scripts, codes and macros used for this study will be provided upon request from the corresponding author.

### Statistics

Data was pre-processed in Excel (Microsoft) and errors and statistics were calculated using Prism (Graphpad). The sample size n refers to the number of culture wells for all data except for electrophysiology data, where n represents the number of single cells. All data were tested for normal distribution: the Mann-Whitney test (for two groups) was applied when sample distributions differed significantly from normal distribution (Shapiro-Wilk test). Data with a normal distribution were tested with the unpaired t-test (for two groups) or ANOVA using the Tukey multiple comparison method (for more than two groups). Data of two cell lines versus passage number or stimulus frequency were tested using two-way repeated-measures ANOVA with the Sidak multiple comparison method. The tests used and the p values are stated in the figure legends. Data are always represented as mean ± standard error of the mean unless otherwise indicated. Significance levels are indicated in the figures in the following manner: *p < 0.05, **p < 0.01, ***p < 0.001, ****p < 0.0001, n.s. not significant.

## Electronic supplementary material

Supplementary information
