# Supplementary material for: On-demand optogenetic activation of human stem-cell-derived neurons
Source: Sci Rep. 2017 Oct 31;7:14450. doi: 10.1038/s41598-017-14827-6 (PMC5663899; doi:10.1038/s41598-017-14827-6)
Supplement: Supplementary file 1 — Supplementary information [file 41598_2017_14827_MOESM1_ESM.pdf]

Supplementary figures and figure legends.

On-demand optogenetic activation of human stem-cell-derived neurons

Simon D Klapper[1*,] Evelyn J Sauter[1*], Anka Swiersy[1], Max AE Hyman[1], Christian Bamann[2], Ernst Bamberg[2] & Volker Busskamp[1#]

1 Center for Regenerative Therapies Dresden (CRTD), Technische Universität Dresden, Dresden, Germany
2 Max Planck Institute of Biophysics, Frankfurt, Germany
* These authors contributed equally to this work.
# Corresponding author: volker.busskamp@tu-dresden.de

Supplementary Figure 1

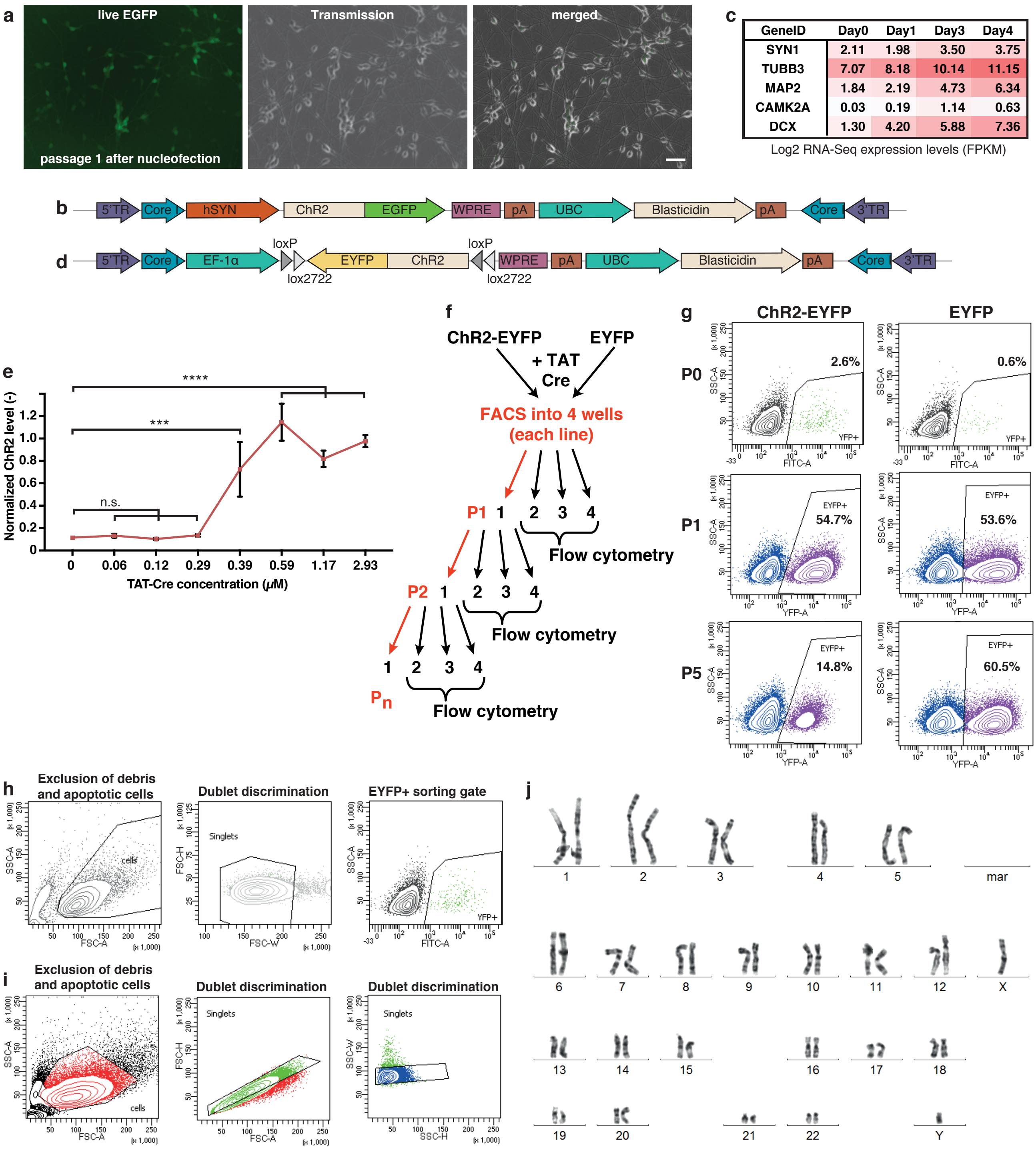

Supplementary Figure 1.

a) Example images of induced iNGN cells expressing genomically integrated ChR2-EGFP under a human synapsin promoter one passage after nucleofection. EGFP fluorescence (green, left), transmission (middle), and merge (right) images are shown. Scale bar, 50 µm.

b) Detailed, linearised map of piggyBac-Syn-CatCh-EGFP-UBC-Bla plasmid.

c) RNA-seq data on expression levels of neuronal markers during neuronal differentiation of iNGN cells, data taken from[5].

d) Detailed, linearised map of piggyBac-eDIO-ChR2-EYFP plasmid.

e) qRT-PCR data showing expression level of ChR2 at different TAT-Cre concentrations. 0.59 µM was the most efficient concentration (0.06 µM: $p = 0.9998$, 0.12 µM: $p = 0.9999$, 0.29 µM: $p = 0.9997$, 0.36 µM: $p = 0.0001$, 0.59 µM: $p = 0.0001$, 1.12 µM: $p = 0.0001$, 2.93 µM: $p = 0.0001$; 1-way ANOVA with Dunnett method versus 0 µM CTRL, $n = 6$ culture wells).

f) Scheme of the flow cytometry approach. After TAT-Cre treatment, cells were sorted for EYFP fluorescence and sequentially passaged 6 times into 4 wells. At every passage, 3 wells were analysed in the flow cytometer, and cells in the remaining well proliferated for the next passage.

g) Example flow cytometry data. At P0, EYFP-positive cells (green) were sorted as the starting population. The raw data show the reduction in EYFP-positive cells (purple) at P1 and P5.

h) Gating strategy used for sorting the EYFP-positive stem cells (see Methods).

i) Gating strategy used to analyse EYFP-positive stem cells 6 sequential passages after sorting (see Methods).

j) Karyogram of Cre-induced and FACS-sorted ChR2-EYFP cells. No chromosomal aberrations could be found.

Scale bar, 50 μm. * $p < 0.05$, ** $p < 0.01$, *** $p < 0.001$, **** $p < 0.0001$, n.s., not significant.

Supplementary Figure 2

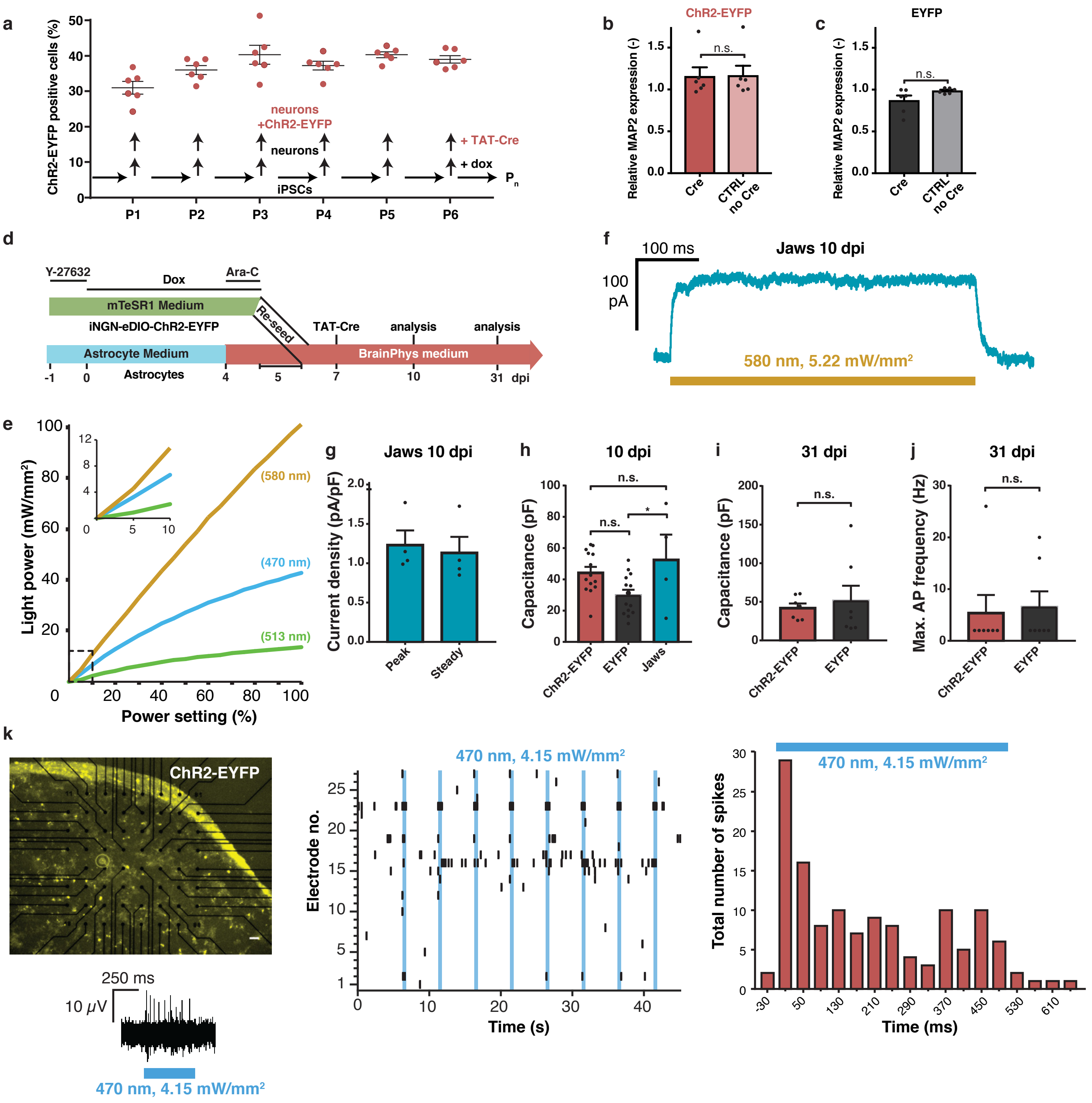

Supplementary Figure 2.

a) Quantification of neurons expressing ChR2-EYFP at 7 dpi. iPSCs were passaged multiple times (P_n) and differentiated into neurons at every passage, followed by TAT-Cre-mediated activation at 4 dpi. Passaging of iPSCs did not affect the efficiency of ChR2-EYFP expression upon TAT-Cre activation, highlighting the stability of the cell line.

b) qRT-PCR data showing MAP2 expression in ChR2-EYFP cells after TAT-Cre treatment compared to no-Cre CTRL. TAT-Cre treatment did not affect MAP2 expression levels ($p > 0.9999$, Mann-Whitney test, $n = 6$ culture wells).

c) qRT-PCR data showing MAP2 expression in EYFP cells after TAT-Cre treatment compared to no-Cre CTRL. TAT-Cre treatment did not affect MAP2 expression levels ($p = 0.0649$, Mann-Whitney test, $n = 6$ culture wells).

d) Scheme of cell culture and treatment outline for electrophysiological recordings.

e) Output light power of Spectra4 light engine measured at the focal plane of the patch-clamp setup. Inset shows range of intensities used.

f) Example traces of voltage-clamp recordings from iNGN-eDIO-Jaws at 10 dpi. Neurons were excited using light pulses of 500 ms.

g) Quantification of peak and steady current density for iNGN-eDIO-Jaws ($n = 4$ cells).

h) Quantification of capacitance at 10 dpi. The capacitance of neurons expressing Jaws-EGFP was different from those expressing EYFP (ChR2 vs. EYFP: $p = 0.0613$, EYFP vs. Jaws: $p = 0.0481$, Jaws vs. ChR2: $p = 0.6417$, 1-way ANOVA with Tukey method, $n = 14$ cells from 4 cover slips from 2 preparations, Jaws: $n = 4$ cells).

i) Quantification of capacitance at 31 dpi. There was no difference between neurons expressing ChR2-EYFP and EYFP ($p = 0.535$, Mann-Whitney test, $n = 7$ cells from 3 cover slips).

j) Quantification of maximum spike frequency at 31 dpi. There was no difference between neurons expressing ChR2-EYFP and EYFP ($p > 0.999$, Mann-Whitney test, $n = 7$ cells from 3 cover slips).

k) Example MEA recording at 17 dpi. Left: iNGN cells expressing ChR2-EYFP (yellow, scale bar, 100 μm) and example trace of filtered data from one electrode upon light stimulus. Middle: raster plot of active electrodes (27 of 59) during eight light stimuli, black bars represent single spikes and blue bars represent 500 ms light stimuli. Right: post-stimulus timing histogram, sum of spikes from all 59 electrodes and 10 light pulses.
